# 1030. Association of Female Sex with Mortality in Patients with *Staphylococcus aureus* Bacteremia: a Systematic Review and Meta-analysis

**DOI:** 10.1093/ofid/ofad500.061

**Published:** 2023-11-27

**Authors:** Annette C Westgeest, Merel M C Lambregts, Rachel E Korn, Maren E Webster, Jackson L Kair, Felicia Ruffin, Joshua B Parsons, Stacey Maskarinec, Samantha Kaplan, Mark G de Boer, Vance G Fowler, Joshua T Thaden

**Affiliations:** Leiden University Medical Center, Leiden, Zuid-Holland, Netherlands; Leiden University Medical Center, Leiden, Zuid-Holland, Netherlands; Duke University Medical Center, Durham, North Carolina; Duke University Medical Center, Durham, North Carolina; Duke University Medical Center, Durham, North Carolina; Duke University Medical Center, Durham, North Carolina; Duke University Medical Center, Durham, North Carolina; Duke University Medical Center, Durham, North Carolina; Duke University Medical Center, Durham, North Carolina; Leiden University Medical Center, Leiden, Zuid-Holland, Netherlands; Duke University Medical Center, Durham, North Carolina; Duke University School of Medicine, Durham, North Carolina

## Abstract

**Background:**

*Staphylococcus aureus* is the leading cause of death due to bacterial blood stream infection. Female sex was a risk factor for mortality in *S. aureus* bacteremia (SAB) in some studies, but not in others. The aim of this systematic review and meta-analysis was to determine whether female sex is associated with mortality in SAB.

**Methods:**

The key question for this systematic review and meta-analysis was as follows: For patients with SAB, is female sex at birth associated with increased mortality? MEDLINE, Embase, and Web of Science were searched from inception to October 31, 2022. We included studies that met all the following conditions: 1) randomized or observational studies evaluating adults with SAB, 2) ≥200 patients, 3) reported mortality at or before 90-days following SAB, and 4) reported mortality stratified by sex (Figure 1). Exclusion criteria included studies on specific subpopulations (e.g. dialysis, ICU, cancer patients), studies that included SAB patients as a subgroup (e.g. patients with bacteremia by any microorganism), and studies using the same cohort as another study included in this review. Both unadjusted and adjusted mortality data, stratified by sex, were extracted.

**Figure 1.**
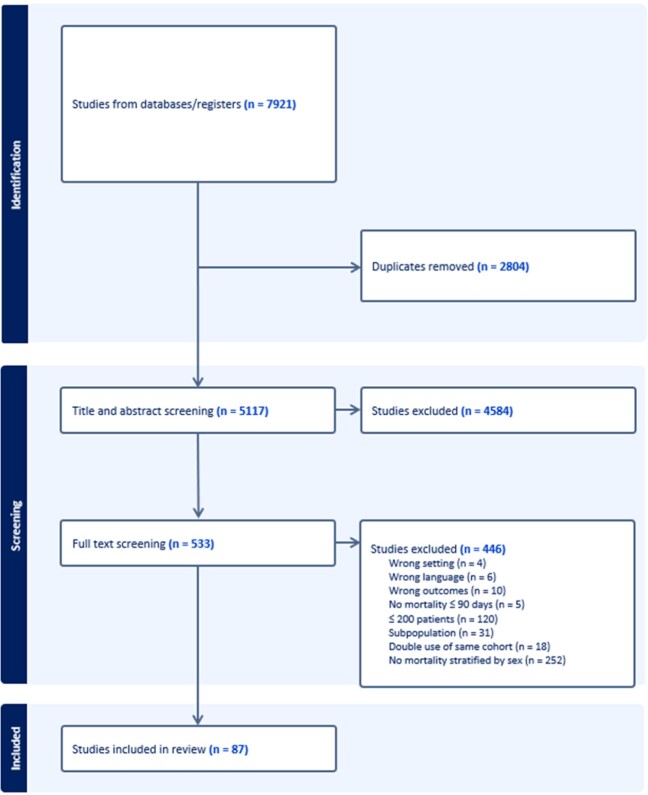
Search flow diagram

**Results:**

From 5117 studies, 87 were included in the final analysis (n=131,356 patients). Study and patient characteristics are summarized in Table 1. Unadjusted mortality data was available from 80 studies (n=108,993 patients) and revealed a small but significant increased mortality in females compared to males (pooled OR 1.12; 95% CI 1.07-1.18; Figure 2). Adjusted mortality data that accounted for additional patient characteristics and treatment variables were available from 31 studies (n=93,683 patients), and revealed a similar increased mortality in females relative to males (pooled aOR 1.19; 95% CI 1.11-1.27; Figure 3). No clear evidence of publication bias was encountered. Sensitivity analysis showed that exclusion of any single study did not influence overall results.

**Table 1.**
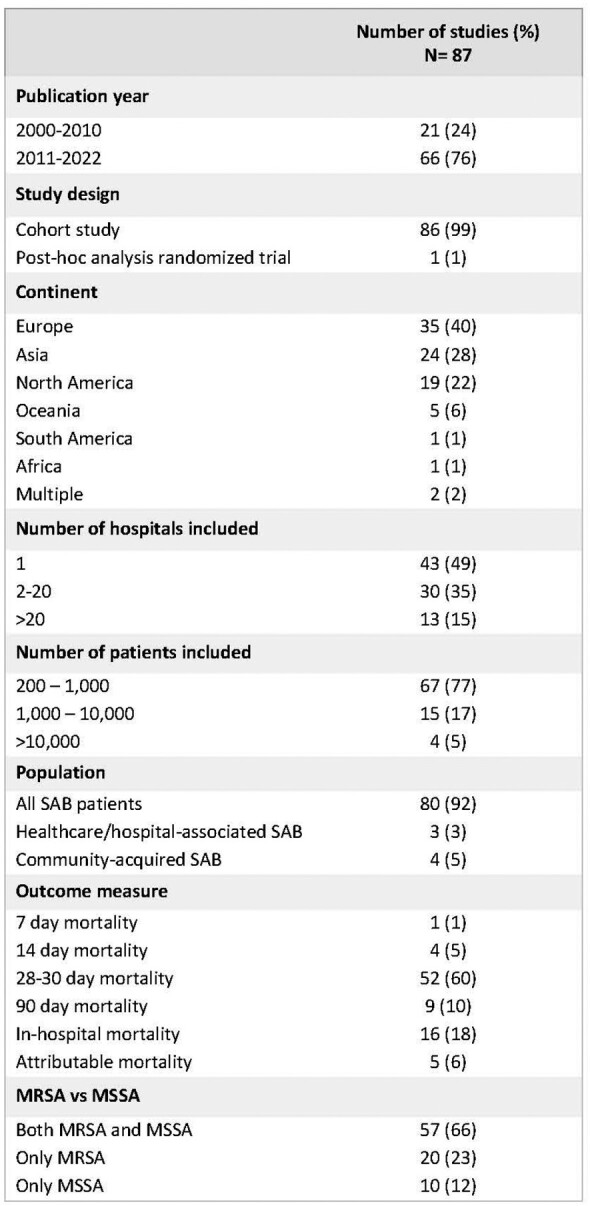
Description of studies

**Figure 2.**
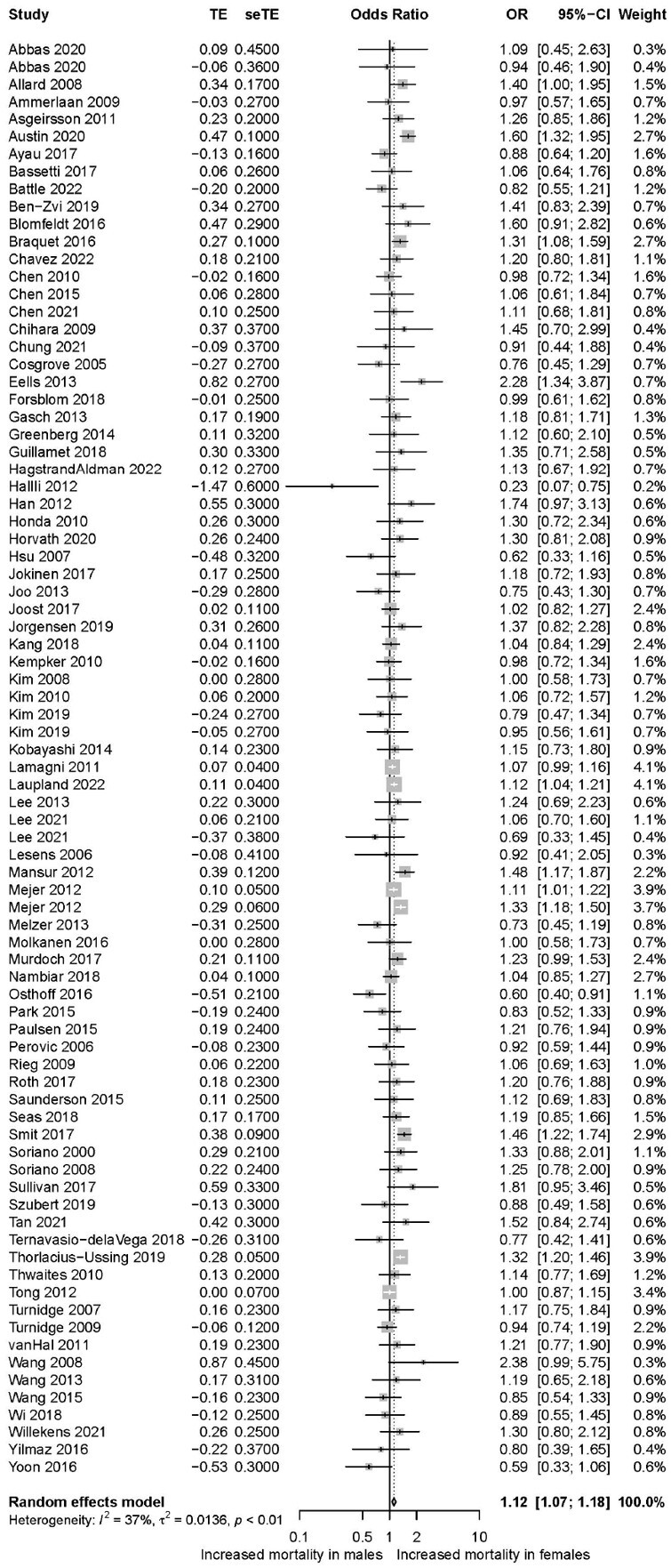
Forest plot of unadjusted mortality in females vs males in SAB

**Figure 3.**
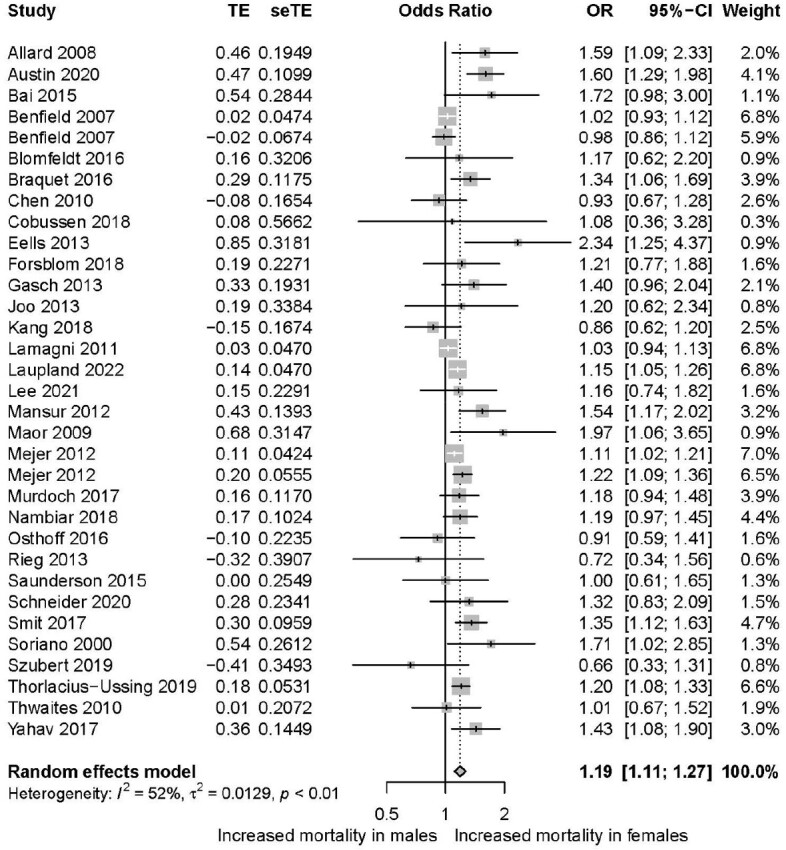
Forest plot of adjusted mortality in females vs males in SAB

**Conclusion:**

Females with SAB have higher mortality than males in both unadjusted and adjusted analyses. Whether this increased mortality is based on biological sex differences rather than social factors has yet to be clarified.

**Disclosures:**

**Stacey Maskarinec, MD, PHD**, National Institutes of Health: Grant/Research Support **Vance G. Fowler, MD, MHS**, Amphliphi Biosciences, Integrated Biotherapeutics; C3J, Armata, Valanbio; Akagera, Aridis, Roche, Astra Zeneca: Advisor/Consultant|Genentech, Regeneron, Deep Blue, Basilea, Janssen;: Grant/Research Support|Infectious Diseases Society of America: Honoraria|MedImmune, Allergan, Pfizer, Advanced Liquid Logics, Theravance, Novartis, Merck; Medical Biosurfaces; Locus; Affinergy; Contrafect; Karius;: Grant/Research Support|Novartis, Debiopharm, Genentech, Achaogen, Affinium, Medicines Co., MedImmune, Bayer, Basilea, Affinergy, Janssen, Contrafect, Regeneron, Destiny,: Advisor/Consultant|Sepsis diagnostic: Patent pending|UpToDate: Royalties|Valanbio and ArcBio: Stock Options **Joshua T. Thaden, MD, PhD**, Resonantia Diagnostics, Inc: Advisor/Consultant

